# Experimental investigations of the human oesophagus: anisotropic properties of the embalmed muscular layer under large deformation

**DOI:** 10.1007/s10237-022-01583-4

**Published:** 2022-04-27

**Authors:** Ciara Durcan, Mokarram Hossain, Grégory Chagnon, Djordje Perić, Lara Bsiesy, Georges Karam, Edouard Girard

**Affiliations:** 1 Zienkiewicz Centre for Computational Engineering, Faculty of Science and Engineering, Swansea University, Swansea, SA1 8EN UK; 2 Univ. Grenoble Alpes, CNRS, UMR 5525, VetAgro Sup, Grenoble INP, TIMC, 38000 Grenoble, France; 3 Laboratoire d’Anatomie des Alpes Françaises, Univ. Grenoble Alpes, Grenoble, France

**Keywords:** Human oesophagus, Mechanical characterisation, Uniaxial tensile deformation, Visco-hyperelasticity, Anisotropy, Stress-softening

## Abstract

The oesophagus is a primarily mechanical organ whose material characterisation would aid in the investigation of its pathophysiology, help in the field of tissue engineering, and improve surgical simulations and the design of medical devices. However, the layer-dependent, anisotropic properties of the organ have not been investigated using human tissue, particularly in regard to its viscoelastic and stress-softening behaviour. Restrictions caused by the COVID-19 pandemic meant that fresh human tissue was not available for dissection. Therefore, in this study, the layer-specific material properties of the human oesophagus were investigated through ex vivo experimentation of the embalmed muscularis propria layer. For this, a series of uniaxial tension cyclic tests with increasing stretch levels were conducted at two different strain rates. The muscular layers from three different cadaveric specimens were tested in both the longitudinal and circumferential directions. The results displayed highly nonlinear and anisotropic behaviour, with both time- and history-dependent stress-softening. The longitudinal direction was found to be stiffer than the circumferential direction at both strain rates. Strain rate-dependent behaviour was apparent, with an increase in strain rate resulting in an increase in stiffness in both directions. Histological analysis was carried out via various staining methods; the results of which were discussed with regard to the experimentally observed stress-stretch response. Finally, the behaviour of the muscularis propria was simulated using a matrix-fibre model able to capture the various mechanical phenomena exhibited, the fibre orientation of which was driven by the histological findings of the study.

## Introduction

The primary function of the oesophagus is to propel swallowed food, in the form of a fluid bolus, from the pharynx into the stomach through a process called peristalsis (Gavaghan 1999). From a mechanical perspective, peristalsis consists of a combination of passive distensions and active contractions of the oesophageal wall, and the interactions of these with the hydrodynamic fluid bolus (Gregersen and Kassab 1996). The mechanical role of the oesophagus can be disrupted by a range of clinical conditions that affect its motor function. Diseases such as diabetes have been found to cause remodelling of the organ in rats which resulted in an increase in the passive stiffness of the oesophageal wall, increasing its resistance to circumferential distension and longitudinal shortening (Yang et al. 2004a). To successfully investigate the pathophysiology that affects the oesophagus’ mechanical function, establishment of the material properties of the healthy human organ is required first (Payan and Ohayon 2017). Knowledge of this would also allow Finite Element models of the oesophagus to be created based on human experimental data, with applications in a range of fields including medical device design and surgical simulations. Medical devices such as oesophageal stents and endoscopy devices benefit from Finite Element models to aid their design process (Peirlinck et al. 2018; Lin et al. 2020; Yim and Sitti 2011). However, currently, the models developed employ parameters based on animal experimental data of the oesophagus (Peirlinck et al. 2018; Lin et al. 2020). In addition, recent developments in endoscopic biopsy devices in the field of robotics require the layer-dependent properties of the human gastrointestinal (GI) organs to be established (Alsunaydih and Yuce 2021). These devices extract diseased tissue samples from the GI tract wall (Simi et al. 2013; Hoang et al. 2020), with one device using the fine-needle capillary technique to pierce through only the inner layer of the wall to retrieve samples of suspected submucosal tumours (Son et al. 2020). For this, a clear differentiation in the mechanical properties of the layers of the GI organs is needed. Further to this, the establishment of the time-dependent properties of the organ are important for surgical simulations in that they add to their realism, thus enhancing the efficacy of the training technique (Taylor et al. 2009). The mechanical properties of the human oesophagus can also be used within tissue engineering to confirm that the material behaviour of the grown tissue matches, or is sufficiently close to, that of the native (Arakelian et al. 2018; Sommer et al. 2013). 

The vast majority of mechanical experimentation on the oesophagus has been carried out ex vivo on animal tissue (Sommer et al. 2013; Stavropoulou et al. 2012; Yang et al. 2004b; Zhao et al. 2007; Gregersen et al. 2008; Saxena et al. 2021; Ren et al. 2021) due to its wider availability and reduced ethical constraints compared to human tissue. For instance, Sommer et al. (2013) investigated the layer-dependent properties of ovine oesophageal tissue using a series of uniaxial tension, biaxial tension, and extension-inflation tests. The tissue exhibited heterogeneous and anisotropic behaviour, with different mechanical properties within the individual layers (the muscularis propria and the mucosa-submucosa ). The rupture strength of the muscularis propria (the muscular layer) was found to be much lower than that of the mucosa-submucosa layer. This is also the case with Stavropoulou et al. (2012) who found the muscularis propria of porcine oesophagi to be less stiff than the mucosa-submucosa layer, associating these findings with the lower collagen content of the muscular layer. Furthermore, Yang et al. (2004b) investigated the properties of rat oesophagi and tested the tissue with the layers intact. In terms of anisotropy, they found the longitudinal direction to be significantly stiffer than the circumferential direction. This is in line with the anisotropic properties established by Sommer et al. (2013) and Stavropoulou et al. (2012). 

In vivo techniques, such as those that measure the distensibility of the tissue (Rao et al. 1995; Patel and Rao 1998; Barlow et al. 2002; Zifan et al. 2021) and include the use of ultrasonic probes (Takeda et al. 2002, 2003; Frøkjaer et al. 2006), have been used to carry out the main proportion of experiments conducted to characterise the human oesophagus. Orvar et al. (1993) found the circumferential wall tension of the organ to increase exponentially with intraluminal pressure, with the same observation being obtained by others in the field (Rao et al. 1995; Patel and Rao 1998; Takeda et al. 2003; Frøkjaer et al. 2006). Patel and Rao (1998) established the non-uniform distribution of stress that appears along the oesophagus, highlighting the relevance of investigating regional discrepancies in regard to its mechanical behaviour. Other studies were found to agree with the findings of Patel and Rao (Frøkjaer et al. 2006; Vanags et al. 2003). The rupture point of the oesophagus, however, cannot be determined from these in vivo experiments due to ethical reasons.

Currently, there is very little data on the ex vivo mechanical behaviour of the human oesophagus (Vanags et al. 2003; Egorov et al. 2002; Tøttrup et al. 1990). Experiments on animal tissue are plentiful, and while animal soft tissues may provide a good representation of how human tissues behave, this data cannot be used to accurately model human tissue to successfully enhance applications in medicine. It is therefore of great interest to perform mechanical experiments on human oesophageal tissue for this purpose. Egorov et al. (2002) conducted a series of experiments on the human GI tract, including the oesophagus. They experimented on fresh human cadavers, tested within 24 hours after death. The tissues were stored in a Euro-Collins solution at 4$^\circ$C prior to testing and their samples were preconditioned. They studied only the distal third of the oesophagus with the layers intact, and tested only in the longitudinal direction. Their main conclusions included that the longitudinal direction of the wall was found to have a high stressibility and exert a maximal stress of 1200 kPa. Note that Egorov and co-workers (Egorov et al. 2002) did not consider the layer-dependent properties of the human oesophagus and only explored the behaviour in one direction. Furthermore, Vanags et al. (2003) investigated the effect of pathology and ageing on the mechanical properties of different regions of the human oesophagus, comparing the results to healthy tissue. The fresh oesophagi, which were studied with their layers intact, were cut into rectangular specimens in both the circumferential and longitudinal directions and subjected to uniaxial tension until rupture. They found that all oesophagi displayed anisotropic behaviour, with higher resistance in the longitudinal direction than the circumferential direction. With age, the Young’s modulus of the tissue wall was found to increase. In addition, the cervical part of the oesophagus displayed the highest ultimate stress and strain when compared with the other two regions (thoracic and abdominal). Note that Vanags and co-workers (Vanags et al. 2003) investigated both directions of loading, but did not consider the layer-dependent properties of the organ.

To the best of the authors’ knowledge, as of yet, the layer-dependent properties of the human oesophagus have not been investigated, particularly in regard to its stress-softening and viscoelastic behaviour. However, due to restrictions caused by the COVID-19 pandemic, fresh human tissue, which is preferential for ex vivo experimentation, was not allowed for mechanical testing. Therefore, this paper aims to provide valuable insight into the layer-specific, direction-dependent cyclic material behaviour of the human oesophagus through mechanical investigation of the embalmed muscularis propria layer. This was carried out through a series of uniaxial cyclic tensile tests conducted in both the longitudinal and circumferential directions with specimens from three different cadavers. The tests were performed at two different strain rates to observe any strain rate-dependent behaviour. Firstly, in Sect. 2 of this study, the anatomy of the oesophagus and experimental procedure are outlined. Next, the experimental findings are systematically presented in Sect. 3. In Sects. 4 and 5, the mechanical behaviour is simulated using a visco-hyperelastic matrix-fibre model and the results are discussed with regard to the histological content of the layer. Finally, in Sect. 6, the findings of the study are summarised and the future work is outlined.

## Experimental methods

### Anatomy of the human oesophagus

**Fig. 1:**
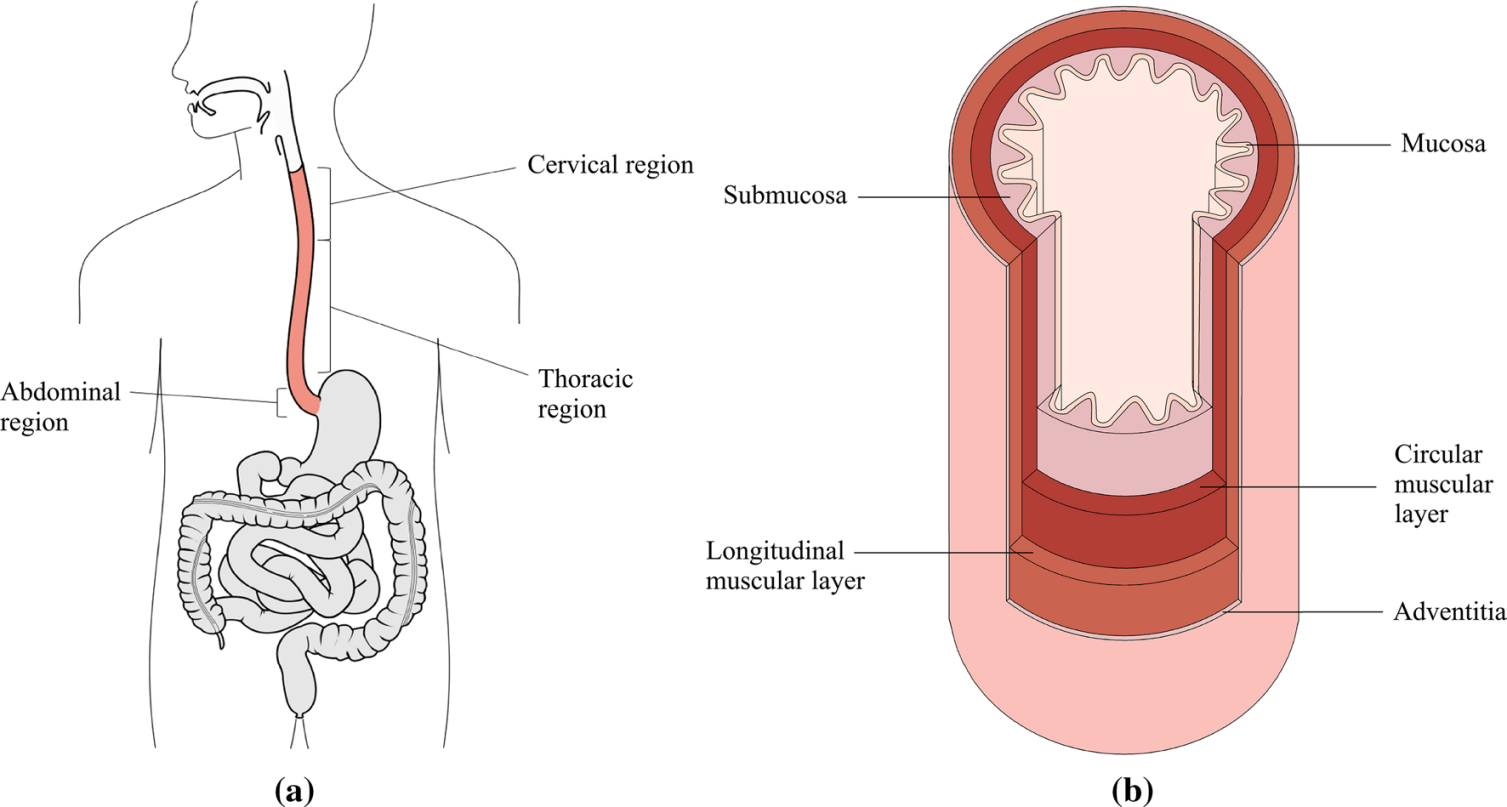
Diagram of the oesophagus showing its position in relation to the rest of the body [modified from (Remesz O (wiki-pl: Orem, commons: Orem), CC BY-SA 2.5: https://creativecommons.org/licenses/by-sa/2.5)] (**a**) and a segment of the organ showing its histological layers (**b**)

The oesophagus is an organ of the digestive system located in the thoracic cavity, as seen in Fig. 1a. It is primarily a mechanical organ consisting of several distinct histological layers, as seen in Fig. 1b. The mucosa is the inner most layer and consists of three separate layers not visible on the diagram: the epithelium, a non-keratinized stratified squamous covering the lumen of the oesophagus; the lamina propria, a thin layer of connective tissue; and the lamina muscularis mucosae, a thin layer of muscle tissue (Ferhatoglu and Kıvılcım 2017). The submucosa is a layer of dense, irregular connective tissue made up of elastin and collagen fibres, containing veins, lymphatics and the submucosal plexus. The muscularis propria layer consists predominantly of muscle fibres arranged longitudinally and circularly. The longitudinal muscle fibres are within a more superficial layer, next to the circular fibres which are situated more deeply, as seen in Fig. 1b. The longitudinal muscle fibres are gathered laterally in the superior portion of the oesophagus; however, they expand and surround all surfaces as one moves inferiorly down the oesophagus, becoming the strongest in the inferior third of the organ (Ferhatoglu and Kıvılcım 2017). Within the superior third of the oesophagus, the circular muscle fibres are elliptical in shape, becoming more circular as one moves inferiorly down the tissue. As well as the orientation, the type of muscle fibres also changes along the length of the organ. The muscle tissue within the first quarter of the oesophagus is striated, with a mix of striated and smooth muscle fibres within the second quarter. The inferior half of the oesophagus consists of smooth muscle only. A thin layer of connective tissue exists between the two muscular layers (the circular/longitudinal junction) containing the vast proportion of collagen fibres present in the muscularis propria (Sommer et al. 2013). The most superficial layer of the oesophagus is the adventitia, which is formed of loose connective tissue that supports the organ’s position in the thorax (Ferhatoglu and Kıvılcım 2017). Once explanted, the oesophagus can be separated into two main layers; the muscularis propria layer and the mucosa-submucosa layer, allowing for their separate mechanical characterisation.

The oesophagus is also made up of several distinct regions. These include the cervical, thoracic and abdominal regions, as seen in Fig. 1a. The cervical region is the superior region and is 5–6 cm in length. At its narrowest point, the cervical region has a luminal diameter of 1.4–1.5 cm, with the remaining regions of the tissue having a luminal diameter of approximately 2 cm. Below the cervical region is the thoracic region, which accounts for the majority of the oesophagus and is 16–18 cm in length. The inferior and smallest region of the tissue is the abdominal region which is 1–2.5 cm in length, depending on the size of the person (Ferhatoglu and Kıvılcım 2017).

### Specimen extraction

Three whole human oesophagi were extracted by means of dissection at the Laboratoire d’Anatomie Des Alpes Françaises, Grenoble, France. Each oesophagus was retrieved from an embalmed cadaver due to restrictions caused by the COVID-19 pandemic, wherein fresh cadavers were not available for dissection. The cadavers were embalmed with a formalin solution (ARTHYL) injected into the carotid artery and drained from the jugular vein, and then preserved in a 4$^\circ$C refrigerated room. All cadavers were required to present a negative COVID-19 test before being allowed for dissection, which could take up to several weeks. The following dissection procedure was used for the explantation of each oesophagus.

A median phreno-laparotomy was performed up to the umbilicus, as well as a left cervical approach following the edge of the sterno-cleido-mastoid muscle. Starting from the stomach, the abdominal part of oesophagus was individualised from the hiatus in the diaphragm. The left triangular ligament, which connects the diaphragm and the posterior surface of the left lobe, was freed, allowing the liver to be reclined. The small omentum was then sectioned off so that the stomach could be freed and hooked up to locate the abdominal oesophagus. The visceral peritoneum in front of the oesophagus was dissected, then a phrenotomy was performed. Dissection of the oesophagus continued in a cranial direction until the pulmonary hilum with its triangular ligament was severed. A right thoracic approach was chosen for the rest of the dissection, allowing the oesophagus to be individualised without being obstructed by the aorta and the heart in the left part of the mediastinum. The right lung was then redirected to the front, and the pulmonary hilum was above the oesophagus. The great azygos vein was also dissected on the right edge of the oesophagus. The abdominal oesophagus was sectioned by making an incision in the fundus of the stomach. The trachea was then sectioned in front of the oesophagus due to it preventing access to the cervical part of the organ. Finally, the cervical oesophagus was sectioned below the pharynx and the whole oesophagus extracted.

This study was performed in compliance with French regulations on postmortem testing, and the protocol approved by a local scientific committee from Université Grenoble Alpes.

### Histology

Prior to mechanical testing, samples for histological analysis were obtained from the oesophagus of Cadaver 1 in the transversal and longitudinal planes of the entire tissue, and the coronal plane of muscularis propria layer. Samples were conserved in formaldehyde, fixed first in formalin 10% during 24 h at 4$^\circ$C and then embedded in paraffin according to usual protocol (Canene-Adams 2013). Sections of 3$\upmu$m were then realised with a microtome Leica RM 2245 (Wetzlar, Germany). The slides were then stained with Haematoxylin Eosin Saffron (HES) to see the nucleic acids and connective tissue (amongst other collagen), with Orcein staining to highlight elastin fibres or with Sirius Red to highlight all types of collagen and muscular fibres.

### Sample preparation

**Fig. 2:**
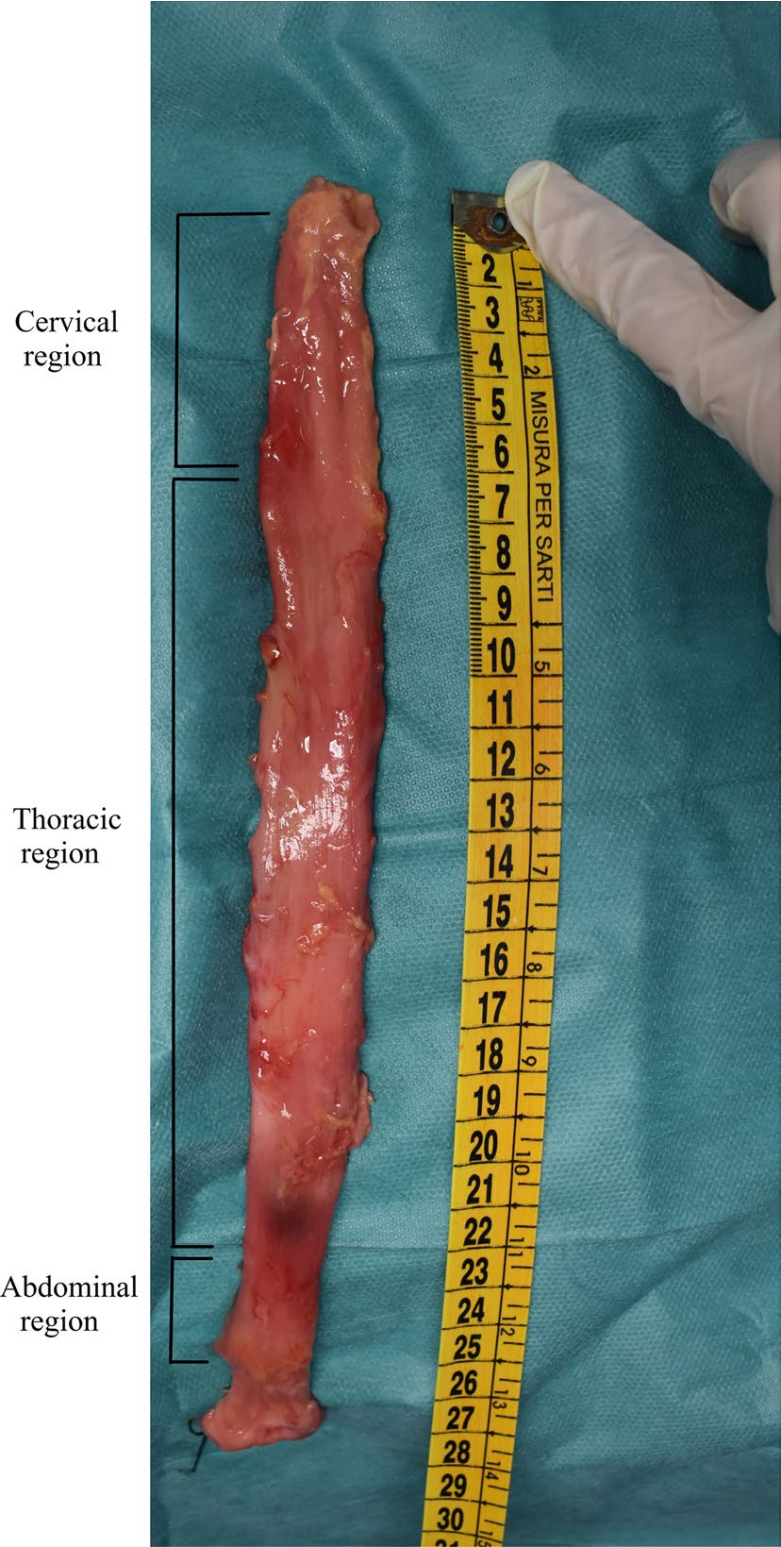
An entire human oesophagus post explantation with its regions labelled

The oesophagi were approximately 27, 26 and 22 cm in size from Cadavers 1, 2 and 3, respectively. In preparation for testing, the oesophagi were cut into their three separate regions (cervical, thoracic and abdominal), as shown in Fig. 2, by cutting along the circumferential direction. The thoracic region was then cleaned by removing any excess connective tissue. Only the thoracic region was used in the study as this region comprises the majority of the organ and the oesophagus’ region-dependent properties were not being investigated.

**Fig. 3:**
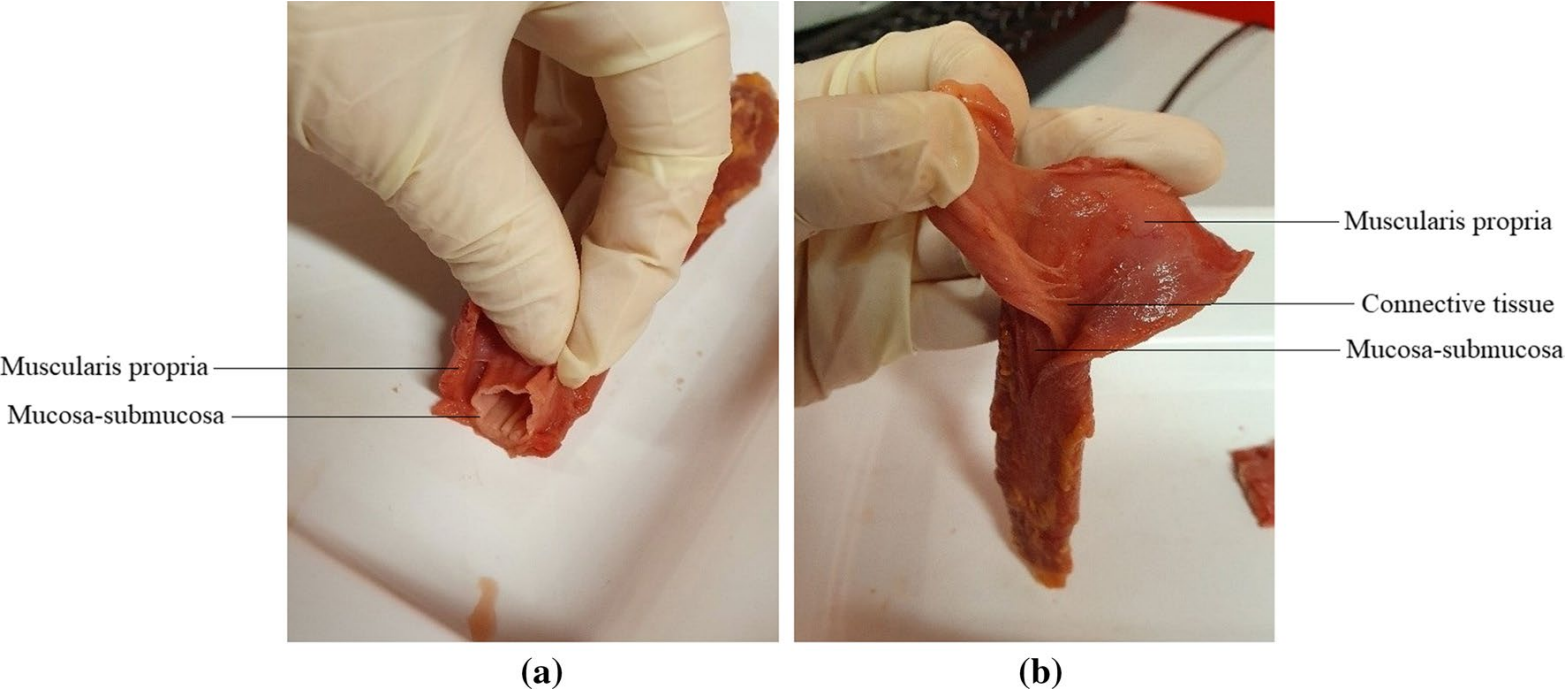
An oesophagus during layer separation showing the distinct layers (**a**) and the connective tissue binding the layers together (**b**)

**Fig. 4:**
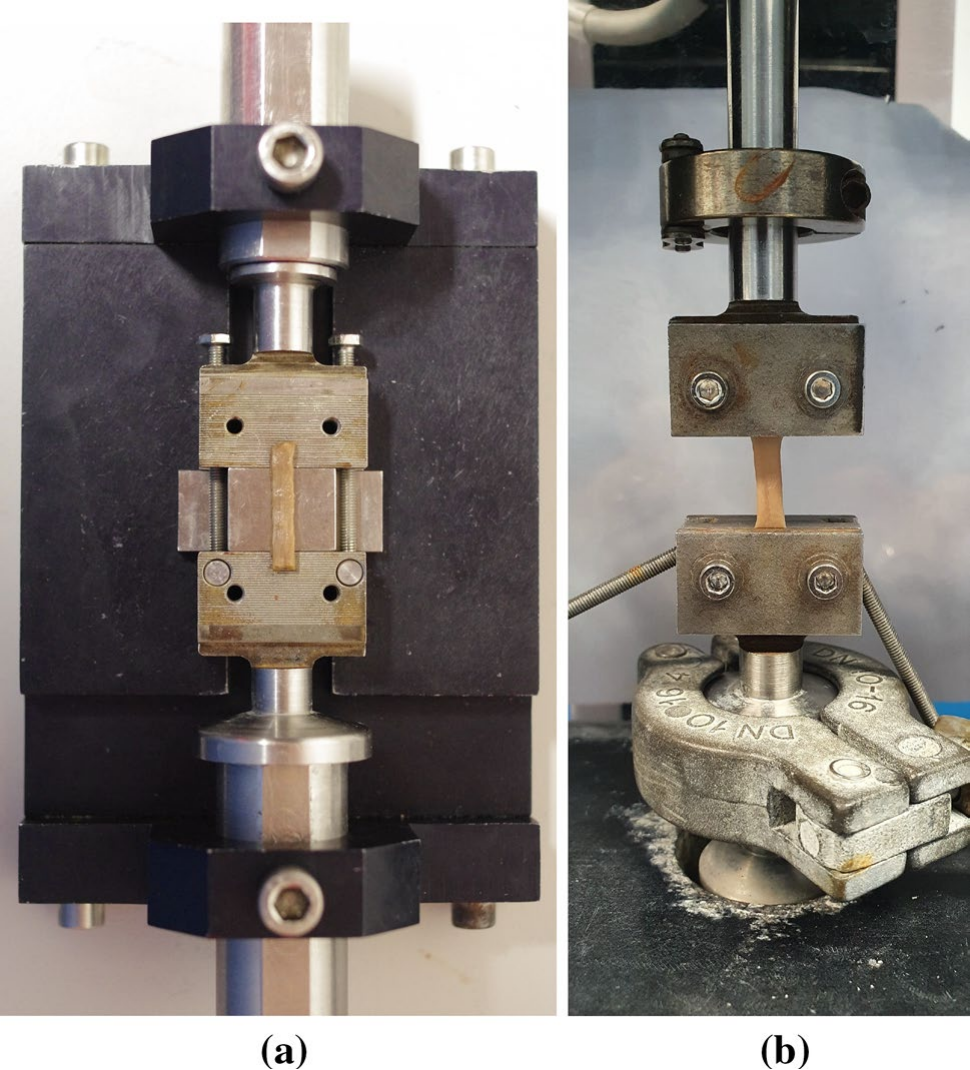
**a** Rectangular sample being loaded between the grips. **b** Sample loaded in the machine

The distinct layers of the oesophagus can be seen in Fig. 3a. The separation of layers was initiated by carefully cutting through the outer, muscular layer, along its longitudinal length. This opening was used to separate the muscularis propria from the mucosa-submucosa through a series of small cuts to the loose connective tissue binding the layers together, as shown in Fig. 3b. The muscular layer was then unravelled and rectangular samples approximately 22.00 mm $\times$ 4.10 mm (length $\times$ width) were cut in both the longitudinal and circumferential directions, as seen in Fig. 4a. Time was taken to cut the samples due to the soft and delicate nature of the tissue, with special care taken to cut them as parallel as possible to the orientation of their respective muscle fibres. The testing was completed within 5 days of explantation, during which the tissue was stored in physiological saline solution (0.9% NaCl) in a 4$^\circ$C refrigerator, with new samples being cut each day. Before testing, the specimens were brought to ambient temperature and were kept moist with saline solution between tests. When analysing the results, there was no correlation to suggest that the length of time between explantation and testing had any influence on the mechanical properties of the samples.

### Experimental setup

The samples were loaded between the grips using a specially designed device, as seen in Fig. 4a. Firstly, each sample was placed centrally on the bottom plates, being held in situ by the support. This step often took some time due to the delicate and moist nature of the tissue causing the sample to stick, proving it difficult to move the sample by the small margins necessary to align it as accurately as possible. Once completed, the top plates of the grips were added and the screws tightened partially, at first, in all four corners to prevent the uneven distribution of the sample within in the grips. The screws were then fully tightened to hold the sample securely in place. A torque limiter set at 0.5 Nm was used for this tightening process to provide consistency and to prevent the sample from slipping during testing. Next, long screws, as seen to the right and left of the sample in Fig. 4a, were tightened to keep the assembly (upper grip, support and lower grip) in place, preventing the sample from incurring any damage while being loaded into the machine. Once setup, the long screws and support were removed, leaving the sample loaded in the traction machine as seen in Fig. 4b. At this point, the thickness and width of the samples were measured using callipers at three separate points along their length and an average was taken. A highly sensitive 25N load cell was fitted to the MTS Criterion model C41 traction machine and was used for all tests due to the comparatively low internal stresses of the muscular layer, as determined by preliminary tests. If the samples buckled while being secured in the grips, the crosshead would be adjusted once the sample was in the machine to reflect its new initial length. To establish the deformation of a sample, an extensometer measured the displacement of the machine’s crosshead. From this, the strain was calculated using the grip-to-grip length of the sample, of which the length-to-width ratio was approximately 4:1, in line with the ASTM standards for uniaxial testing (ASTM 2013). The machine and test parameters were controlled and inputted using the MTS TestSuite user interface software.

### Mechanical characterisations

The experimental strain is expressed in terms of stretch, $\lambda$, which relates to nominal strain by $\varepsilon =\lambda - 1$. Stretch is defined as $\lambda = \frac{l}{l_0}$, where *l* and $l_0$ are the current and initial lengths of the sample, respectively. The strain rates are described in units of percentage deformation per second (% ${\text{s}}^{-1}$). Similarly, the stress is expressed as the nominal stress (i.e., first Piola–Kirchhoff stress), which is defined as:

1 $$\begin{aligned} P = \frac{F}{A_0} \end{aligned}$$

where *F* is the applied force and $A_0$ is the original, undeformed cross-sectional area.

Uniaxial tension tests, wherein the lengths of the samples are substantially larger than their widths and the force exerted along a single axis parallel to their length, were conducted to observe the material response of the tissue. The tests were carried out in the form of increasing stretch level cyclic tests with two cycles per stretch level to study the material’s viscoelastic behaviour and to observe its approximate preconditioned response (Hossain and Liao 2020; Hossain et al. 2020, 2012; Masri et al. 2018). Stretch levels in increments of 0.1 from 1.1 to 1.7 were utilised to investigate a range of deformations. This range was determined from in vivo tests performed by Takeda et al. (2002) who, by isobaric distension, found the circumferential stretch of the oesophagus to be in the range of 1.15–1.70. The tests were conducted in both the longitudinal and circumferential directions to observe the effect of loading direction, and were carried out at two different strain rates, 1% ${\text{s}}^{-1}$ and 10% ${\text{s}}^{-1}$, to study any strain rate effects. All tests were performed at ambient temperature. For each cadaver, 5–11 tests per direction, per strain rate were conducted to ensure reproducible results. A new sample was used for each test, and all tests were carried out until 1.7 stretch or until rupture, i.e. if the sample ruptured before reaching the 1.7 stretch level, the test was terminated.

## Results

### Histological analysis of the muscularis propria layer of the human oesophagus

**Fig. 5:**
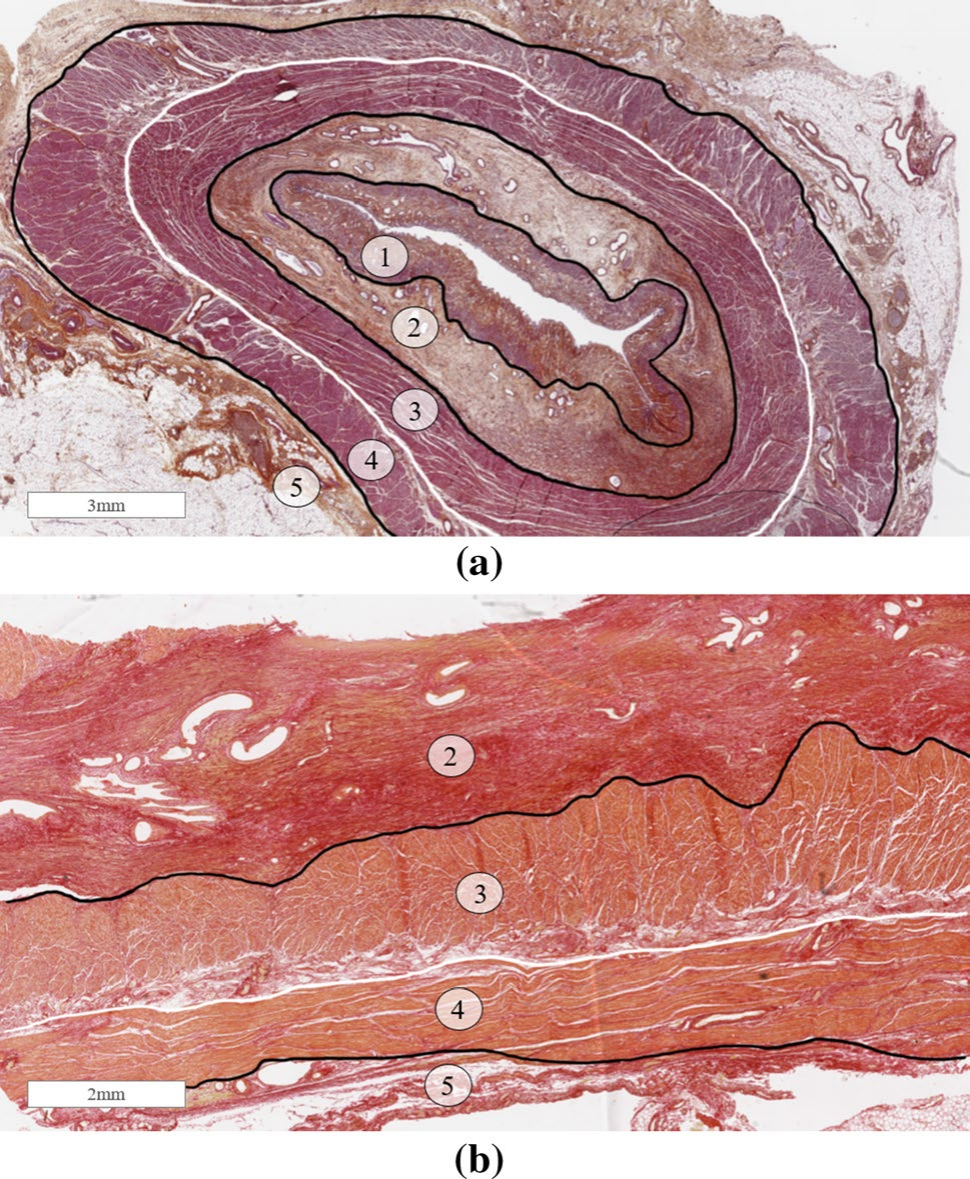
Haematoxylin Eosin Saffron staining in the transversal plane (**a**) and Sirius Red staining in the longitudinal plane (**b**) showing the mucosa (**1**), submucosa (**2**), the circular muscle fibres of the muscularis propria (**3**), the longitudinal muscle fibres of the muscularis propria (**4**) and the adventitia (**5**)

In the transversal plane, the four layers of the oesophagus (outlined in Sect. 2.1) are clearly visible and can be seen in Fig. 5a. These layers are also evident in the longitudinal plane, as shown in Fig. 5b. Compared to the mucosa-submucosa, the muscularis propria has a lower concentration of collagen and elastin fibres. Overall, there are more collagen fibres than elastin fibres in the fibrous composition of the muscularis propria (both circular and longitudinal layers). In the inner circular layer, the collagen fibres form a mesh orthogonal to the axis of the oesophagus. Fibres have a transversal orientation with some oriented along the muscle cells and others towards the lumen. Elastin fibres have a similar orientation in this layer. The fibres are mostly concentrated at the circular/longitudinal junction of the muscularis propria, organised mainly along the axis of the oesophagus. The outer longitudinal layer has its collagen fibres mainly oriented in the longitudinal direction, with few fibres oriented towards the lumen. The elastin fibres have a longitudinal orientation also, but with a very wavy appearance. The elastin fibres seem to be more abundant in the outer longitudinal layer than in the inner circular layer. The distribution of collagen and elastin fibres within the muscularis propria are summarised in Table 1.

**Table 1:** Distribution of collagen and elastin in the muscularis propria; +, low density; ++++, high density

Layer	Collagen	Elastin
Circular muscle	++	+
Circular/longitudinal junction	++++	+++
Longitudinal muscle	++	++

### Demographics and variations in experimental samples

The oesophagi were retrieved from three embalmed cadavers fixed in formalin, the demographics of which can been seen in Table 2. Dimensions such as thickness can vary from sample to sample due to the variable nature of biological tissues. The mean and standard deviations of the sample dimensions for each direction and strain rate can be seen in Table 3.

**Table 2:** Patient demographics and time of embalming

Cadaver	Sex	Height [cm]	Weight [kg]	Age [years]	Time of embalming [days]
1	Male	180	73	90	29
2	Female	153	40	97	71
3	Female	140	40	101	40

**Table 3:** Mean ± population standard deviation of sample dimensions

	1% s$^{-1}$		10% s$^{-1}$	
	Width [mm]	Thickness [mm]	Width [mm]	Thickness [mm]
Longitudinal	4.04 ± 0.30	1.21 ± 0.41	4.16 ± 0.26	1.09 ± 0.29
Circumferential	4.06 ± 0.25	1.42 ± 0.50	4.10 ± 0.25	1.32 ± 0.37

### Reproducibility in stress–strain data and statistical analysis

**Fig. 6:**
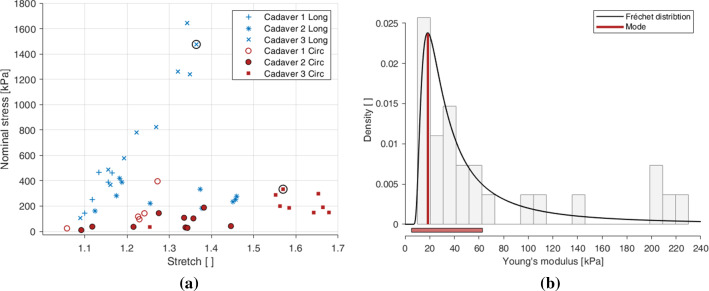
Rupture points of each test conducted at 10% ${\text{s}}^{-1}$ highlighting the dispersion between cadavers and the longitudinal and circumferential directions (**a**), and the combined histogram and probability distribution graph showing the dispersion of Young’s modulus for the 10% ${\text{s}}^{-1}$ circumferential experimental results across three cadavers displaying a significant ($p=0.972$) Fréchet distribution at $\alpha =0.05$ with a mode (range) of 18.5 (5.2–62.9) (**b**). The vertical red line in (**b**) shows the mode of the probability distribution, while the horizontal red bar depicts the range

When comparing the stress–strain behaviour of the tests within a single test condition (direction and strain rate), there was a high amount of dispersion. This is demonstrated in Fig. 6a which shows the point of complete rupture, in terms of stress and stretch, for each test conducted at 10%$\, s^{-1}$, highlighting the differences between cadavers and directions. Rupture of the sample was defined as irreversible macrostructural damage, visible on the stress–strain graph as a sudden reduction in stress. The samples ruptured in various locations, including in the middle of the sample, at the grip location, or a combination of both.

Due to the dispersion of the data, a statistical approach was undertaken to retrieve the most representative behaviour of the embalmed muscular layer of the human oesophagus (per direction and strain rate) to use for analysis and constitutive modelling. Firstly, the Young’s modulus for each test was calculated by taking the gradient of the first loading curve of the first cycle from 1.00 to 1.01 stretch. The Young’s modulus, *E*, is defined as:

2 $$\begin{aligned} E = \frac{P}{\varepsilon } \end{aligned}$$

where *P* is the nominal stress and $\varepsilon$ is the nominal strain as described in Sect. 2.6. The number of tests per direction, per strain rate and per cadaver can be seen in Table 4. A histogram with 20 bins was then plotted for each test condition with density on the y-axis and Young’s modulus on the x-axis, as seen in Fig. 6b. The histogram for each test condition presented a right skewed distribution of Young’s moduli, therefore a number of non-normal distributions were chosen to test against a null hypothesis, including chi-squared distribution (Kissell and Poserina 2017), gamma distribution (Thom 1958) and Fréchet distribution (Harlow 2002). The null hypothesis for a specific distribution, direction and strain rate was,“The Young’s modulus of the [specific] direction of the muscular layer of the embalmed human oesophagus tested at a strain rate of [specific]% ${\text{s}}^{-1}$ is distributed according to the [specific] distribution.”. All statistical tests were carried out using R Statistical Software and with a significance level, $\alpha$, of $\alpha =0.05$, meaning that the null hypothesis is rejected if $p<0.05$.

The null hypotheses of all test conditions were retained for the gamma and Fréchet distributions as $p>0.05$. The Fréchet distribution, however, was chosen as it is the most appropriate for the data set (Harlow 2002), with p-values of ($p=0.367$) for the 1% ${\text{s}}^{-1}$ longitudinal results, ($p=0.748$) for the 10% ${\text{s}}^{-1}$ longitudinal results, ($p=0.808$) for the 1% ${\text{s}}^{-1}$ circumferential results, and ($p=0.972$) for the 10% ${\text{s}}^{-1}$ circumferential results.

The mode of the Fréchet distribution gives the most likely value of the Young’s modulus of the population, and the range, in this circumstance, provides the range in which 70% of all Young’s moduli of the population reside. To provide the most representative behaviour for the following analysis and constitutive modelling, the test with the Young’s modulus closest to the mode of the Fréchet distribution for each test condition was selected. The Young’s moduli of the 1% ${\text{s}}^{-1}$ longitudinal experimental results had a mode (range) of 32.2 (6.0–266.0), the 10% ${\text{s}}^{-1}$ longitudinal experimental results had a mode (range) of 38.6 (7.0–339.0), the 1% ${\text{s}}^{-1}$ circumferential results had a mode (range) of 16.1 (3.7–81.5) and the 10% ${\text{s}}^{-1}$ circumferential results had a mode (range) of 18.5 (5.2–62.9), as seen in Fig. 6b. The circled rupture points in Fig. 6a correspond to the selected 10% ${\text{s}}^{-1}$ tests in each direction, the cyclic stress-strain data of which will be subsequently presented. The Young’s moduli that have been calculated here have only been used to assess the variations within the results and to extract a representative curve for each test condition, and have not been used explicitly in the modelling of the layer’s stress-stretch response.

**Table 4:** Number of tests per direction, per strain rate, per cadaver

Direction	Strain rate	Cadaver	Tests	Total
Longitudinal	1%/s	1	5	n = 25
		2	10	
		3	10	
	10%/s	1	5	n = 25
		2	10	
		3	10	
Circumferential	1%/s	1	5	n = 26
		2	10	
		3	11	
	10%/s	1	5	n=26
		2	10	
		3	11	

### Presentation of experimental results

The cyclic tests were performed with two cycles per stretch level, and both cycles have been presented here. As can be seen in Fig. 6a, the rupture of the samples occurred at different stretches. With that in mind, only the full cycles of each result have been presented. For instance, the 10% ${\text{s}}^{-1}$ longitudinal sample ruptured during the 1.4 stretch level cycle between 1.3 and 1.4 stretch (Fig. 6a), and therefore only the results up until the 1.3 stretch level are presented for this test.

### Anisotropic response

**Fig. 7:**
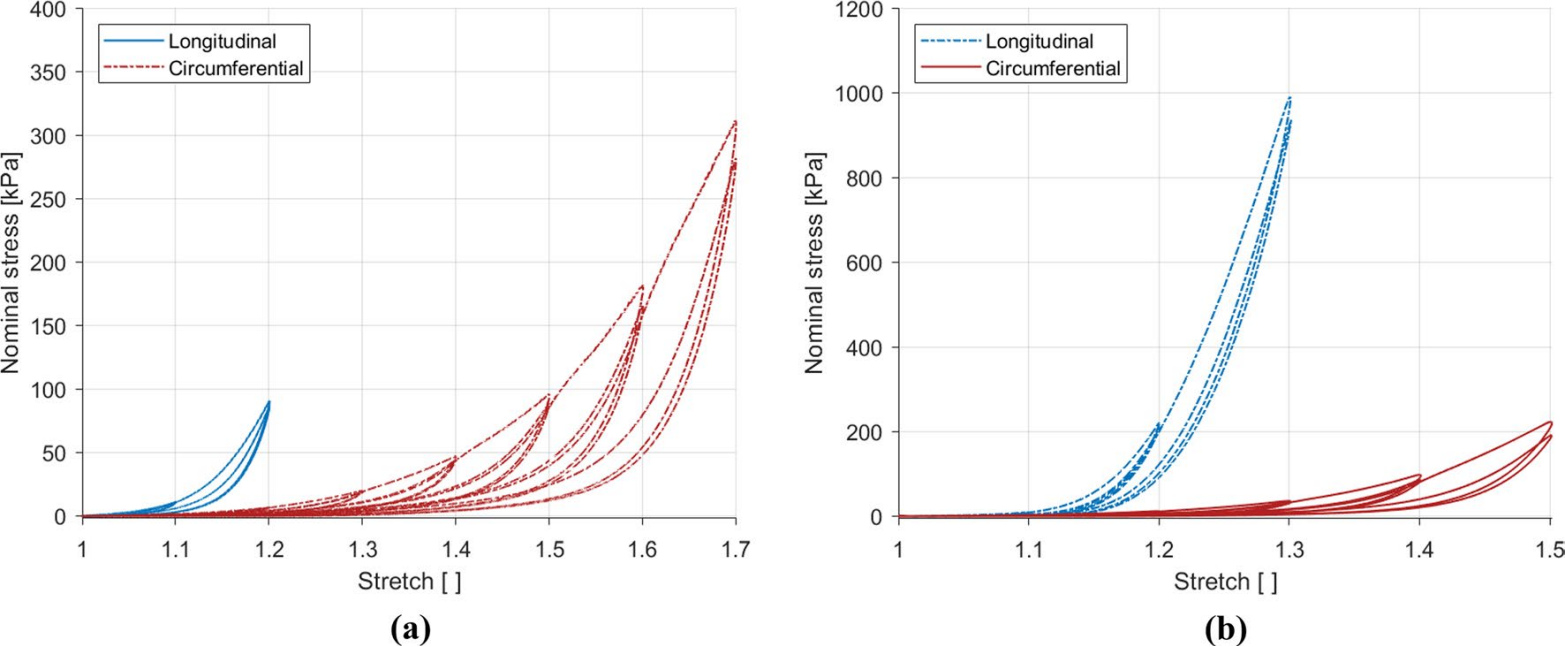
Effects of the direction of loading on the results at 1% s$^{-1}$ (**a**) and 10% s$^{-1}$ (**b**) of the embalmed muscularis propria layer

The results comparing the loading directions for the two strain rates are presented in Fig. 7. The embalmed human oesophageal muscular layer displays anisotropic properties at both strain rates. The stiffness was higher in the longitudinal direction than the circumferential direction for the 1% s$^{-1}$ and 10% s$^{-1}$ tests, while the samples in the longitudinal direction ruptured earlier than those in the circumferential direction.

### Strain rate-dependent behaviour

**Fig. 8:**
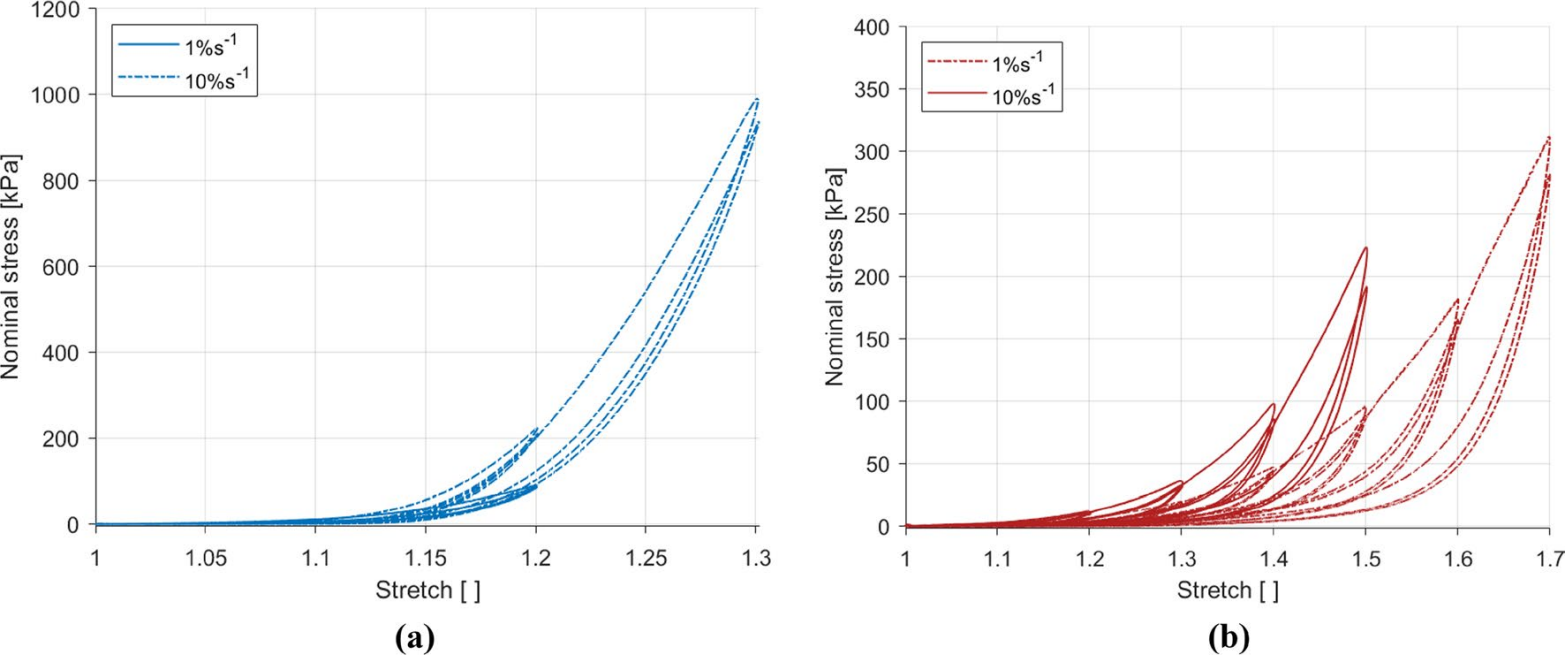
Effects of loading rate on the results in the longitudinal direction (**a**) and the circumferential direction (**b**) of the embalmed muscularis propria layer

Figure 8 compares the strain rates for the longitudinal and circumferential directions. The strain rate was found to affect the stiffness in the longitudinal and circumferential directions, with an increase in the strain rate resulting in an increase in the stiffness. Hysteresis was also found to be greater for the 10% s$^{-1}$ results compared to the 1% s$^{-1}$ results in both directions. At comparable stretches, the strain rate effect was greater in the longitudinal direction than in the circumferential direction.

### Permanent deformations

**Fig. 9:**
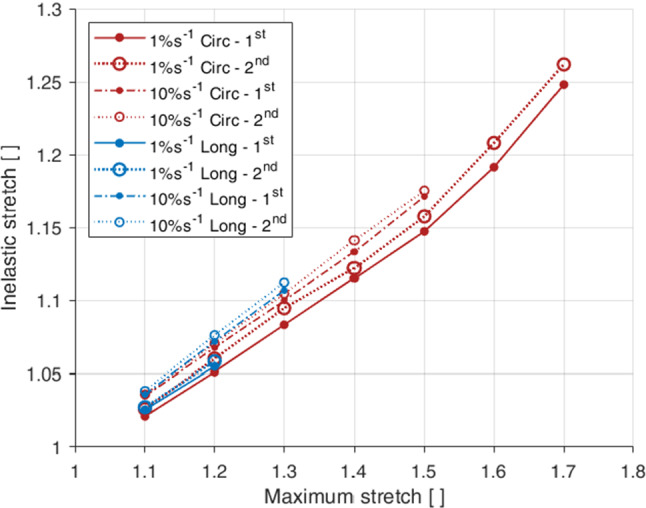
Permanent set in each loading direction corresponding to the maximum stretch of the previous load cycle, for both the first and second cycles at both strain rates (1% s$^{-1}$ and 10% s$^{-1}$)

Permanent deformations describe the residual, inelastic strains present in a material once it has been unloaded. The permanent deformations, often referred to as permanent set, of each test condition compared to the previous maximum stretch can be seen in Fig. 9, including also a comparison between the first and second cycle for a single stretch level. Permanent set was found to increase with an increase in maximum stretch for all directions, strain rates and cycles. The gradient of increase was similar for both the longitudinal and circumferential directions. These inelastic strains were found to be greater for the second cycle compared to the first cycle for all directions and strain rates. They were also found to be greater for the 10% s$^{-1}$ tests than the 1% s$^{-1}$ tests for both the longitudinal and circumferential directions, thus implying strain rate effects within the permanent set.

## Constitutive modelling

In this section, we aim to constitutively model the behaviour of the embalmed muscularis propria layer. In the experimental data, we observed a range of phenomena including anisotropy, strain rate-dependent behaviour, hysteresis, stress-softening and permanent set. The cyclic behaviour of soft materials can be modelled in a variety of ways, including pseudo-elastic based models (Ren et al. 2021; Fereidoonnezhad et al. 2016; Peña et al. 2009; Ehret and Itskov 2009) and those derived from continuum damage mechanics (Maher et al. 2012; Balzani et al. 2012; Schmidt et al. 2014; Rodríguez et al. 2006). Here, we intend to simulate the experimentally observed behaviour using a unique formulation of an anisotropic, visco-hyperelastic matrix-fibre model with an added damage function.

### Anisotropic matrix-fibre model with damage

**Fig. 10:**
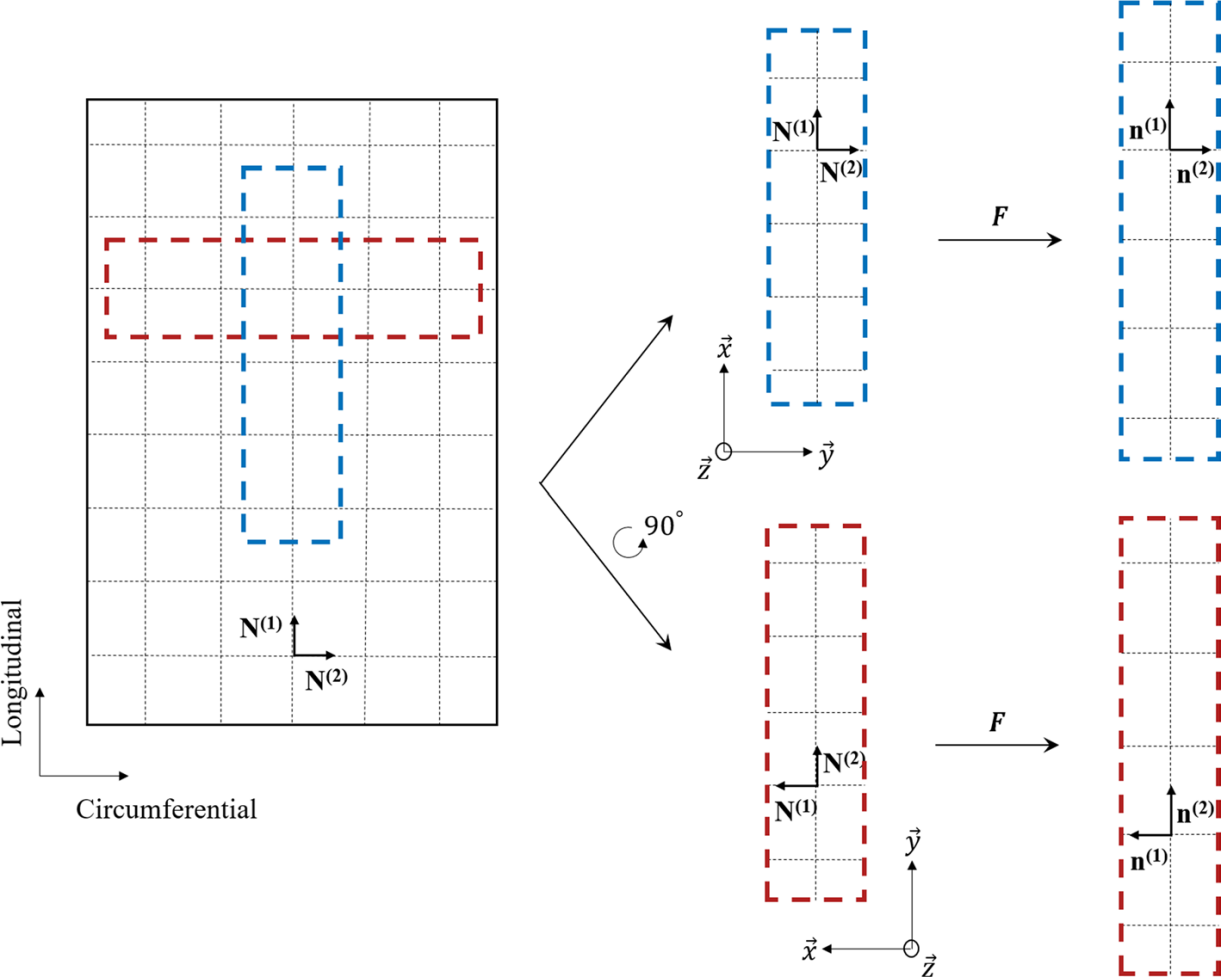
Drawing to illustrate the fibre orientation of the muscularis propria layer of the human oesophagus based on the histological observations outlined in Sect. 3.1

Firstly, an orthotropic model is used to capture the anisotropy. Here, the matrix of the tissue has been considered as purely elastic and isotropic, with the anisotropy, viscoelasticity and damage originating from the fibres and their predominant orientations. This is due to collagen being posited as one of the main components contributing to the tensile strength and viscoelastic behaviour of soft tissues (Aziz et al. 2016; Li et al. 2005; Yang et al. 2006a). For the muscular layer, the fibres are composed of the collagen networks surrounding and connecting the muscle fibres. From our histological analysis, as outlined in Sect. 3.1, the fibres were observed to sit predominantly in the same direction as their respective muscle fibres. Therefore, the collagen fibres of the muscular layer are considered to be orientated orthogonal to each other in the longitudinal and circumferential directions, as demonstrated in Fig. 10. This is contrary to similar animal studies who modelled the muscular layer using two families of collagen fibres both orientated at an angle to the circumferential direction (Sommer et al. 2013; Sokolis 2013), i.e. not perpendicular and parallel as is adopted here. For the model, the stress can either be described in terms of the undeformed configuration, i.e. the reference configuration, or the deformed configuration. Here, either will be used depending on convenience. The stress in the undeformed configuration is described by the second Piola–Kirchhoff (PK) stress tensor which can be defined for incompressible, anisotropic materials as:

3 $$\begin{aligned} \varvec{S} = -p\varvec{C}^{-1} + 2 \frac{\partial W_0}{\partial {I_1}}\varvec{I} + 2 \sum _{i=1,2}^{}{I_4^{\left( i\right) }\frac{\partial W_1^{\left( i\right) }}{\partial {I_4^{\left( i\right) }}}}\left[ \varvec{N}^{\left( i\right) }\otimes \varvec{N}^{\left( i\right) }\right] \end{aligned}$$

where *p* is the hydrostatic pressure used to impose the incompressibility constraint, $\varvec{C}$ = $\varvec{F}^T\,\varvec{F}$ is the right Cauchy-Green tensor , $\varvec{F}$ is the deformation gradient tensor, $\varvec{I}$ is the identity tensor, $\varvec{N}^{\left( i\right) }$ is the direction of each set of fibres in the undeformed configuration, and $W_0$ and $W_1^{\left( i\right) }$ are the strain energy functions (SEFs) of the matrix and fibres, respectively, in which $I_1$ and $I^{(i)}_{4}$ are defined as:

4 $$\begin{aligned} I_1 = \text {tr}(\varvec{C}); \quad I^{(i)}_{4}= \varvec{C} :\left[ \varvec{N}^{(i)}\otimes \varvec{N}^{(i)}\right] . \end{aligned}$$

The stress in the deformed configuration is described by the Cauchy stress tensor, which can be defined for incompressible, anisotropic materials by:

5 $$\begin{aligned} \varvec{\sigma } = -p\varvec{I} + 2 \frac{\partial W_0}{\partial {I_1}}\varvec{b} + 2 \sum _{i=1,2}^{}{I_4^{\left( i\right) }\frac{\partial W_1^{\left( i\right) }}{\partial {I_4^{\left( i\right) }}}}\left[ \varvec{n}^{\left( i\right) }\otimes \varvec{n}^{\left( i\right) }\right] \end{aligned}$$

where $\varvec{b}$ = $\varvec{F}\,\varvec{F}^T$ is the left Cauchy-Green tensor and $\varvec{n}^{\left( i\right) }$ is the direction of each set of fibres in the deformed configuration. $\varvec{n}^{(i)}$ = $\varvec{F}\,\varvec{N}^{(i)}$ captures the fibre orientation in the deformed state at any given time.

Next, the damage is modelled using a stress-softening evolution function developed by Rebouah et al. (2013). This function, $\chi$, is also able to capture the permanent set of a material and is applied here to the fibre portion of the equation. Thus, the Cauchy stress tenor becomes:

6 $$\begin{aligned} \varvec{\sigma } = -p\varvec{I} + 2 \frac{\partial W_0}{\partial {I_1}}\varvec{b} + 2 \sum _{i=1,2} {\chi ^{\left( i\right) }\left( I_4^{\left( i\right) },I_4^{\left( i\right) max}\right) I_4^{\left( i\right) }\frac{\partial W_1^{\left( i\right) }}{\partial {I_4^{\left( i\right) }}}}\left[ \varvec{n}^{\left( i\right) }\otimes \varvec{n}^{\left( i\right) }\right] . \end{aligned}$$

The stress-softening function considers the difference between the current stretch and the previous maximum stretch, thus capturing the history-dependent behaviour of the material. The function was applied to soft tissues by Rebouah and Chagnon (2014) and is described as:

7 $$\begin{aligned} \chi ^{\left( i\right) }\left( I_4^{(i)},I_4^{(i)\max }\right) =1-\eta _{\text{m}}^{\left( i\right) } \left[ \frac{I_4^{(i)\max }-I_4^{(i)}}{I_4^{(i)\max }-1}\right] ^{\beta ^{\left( i\right) }} \end{aligned}$$

where $\eta _{\text{m}}$ and $\beta$ are dimensional parameters, and $I_4^{(i)\max }$ is the maximum value of $I_4^{(i)}$ for each direction throughout the whole history of the material.

**Fig. 11:**
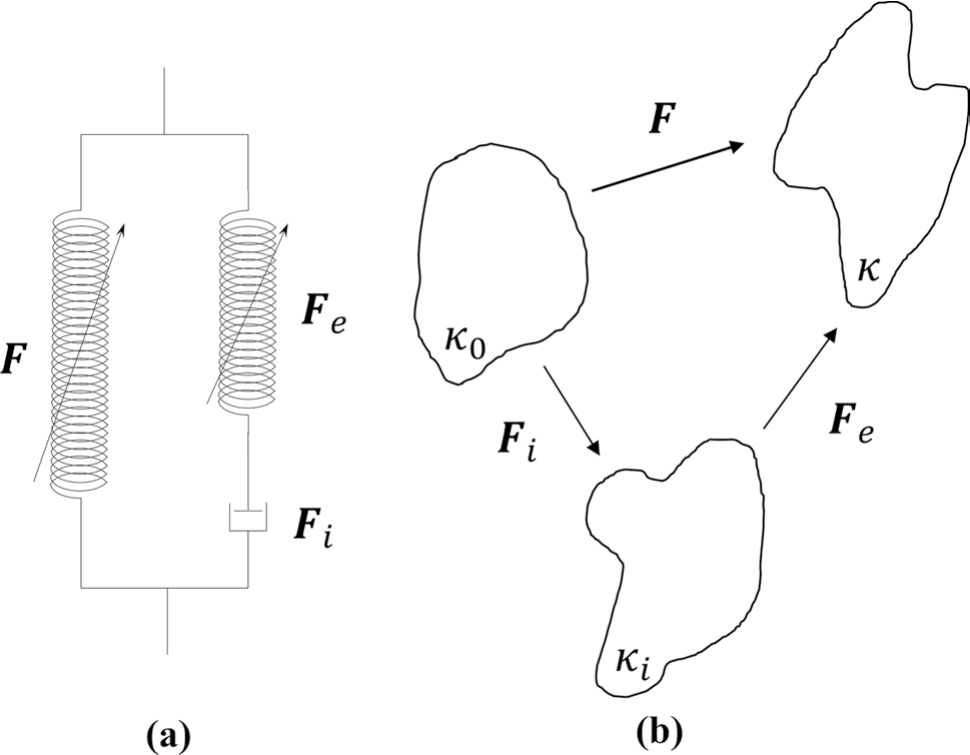
Rheological representation of the viscoelastic model (**a**) and the decomposition of the deformation gradient tensor $\varvec{F}$ (**b**), where $\kappa _0$ is the undeformed configuration, $\kappa _i$ is the intermediate configuration and $\kappa$ is the final configuration

To capture the viscoelasticity of the muscularis propria, an internal variable-based model advocated by Petiteau et al. (2013) is implemented. This viscoelastic contribution is applied only to the fibres, and can be depicted using a spring-dashpot analogy in the form of a generalised Maxwell model, as seen in Fig. 11a. It can be seen that in the left, elastic branch of the schematic diagram (Fig. 11a) the previously described stress-softening function has been added. This branch is captured by the overall deformation gradient tensor of the material. The right, inelastic branch can be described by a multiplicative decomposition of the deformation gradient tensor into an elastic part, $\varvec{F}_e$, with stress-softening and an inelastic part, $\varvec{F}_i$. A visual representation of this can be seen in Fig. 11b, in which $\varvec{F} =\varvec{F}_e\varvec{F}_i$.

The material and each deformation is assumed to be incompressible, and so the volume between $\kappa _0$ and $\kappa _i$ remains constant. Therefore, the SEF of the fibres can be written as:

8 $$\begin{aligned} W_{\text {fibres}}(\varvec{F},\varvec{F}_e) = W_1(\varvec{F}) + W_2(\varvec{F}_e) \end{aligned}$$

where $W_1$ is associated with the deformation between $\kappa _0$ and $\kappa$, and $W_2$ is the SEF involved in the deformation between $\kappa _i$ and $\kappa$. Therefore, the Cauchy stress becomes:

9 $$\begin{aligned} \varvec{\sigma } = -p\varvec{I} + 2 \frac{\partial W_0}{\partial {I_1}}\varvec{b} + 2 \sum _{i=1,2}^{}{\chi ^{\left( i\right) }\left[ I_4^{\left( i\right) }\frac{\partial W_1^{\left( i\right) }}{\partial {I_4^{\left( i\right) }}}\left[ \varvec{n}^{\left( i\right) }\otimes \varvec{n}^{\left( i\right) }\right] + I_{4,e}^{\left( i\right) }\frac{\partial W_2^{\left( i\right) }}{\partial {I_{4,e}^{\left( i\right) }}}\left[ \varvec{n}_e^{\left( i\right) }\otimes \varvec{n}_e^{\left( i\right) }\right] \right] } \end{aligned}$$

where

10 $$\begin{aligned} I^{(i)}_{4,e}= \varvec{C}_e :\left[ \varvec{N}^{(i)}\otimes \varvec{N}^{(i)}\right] \end{aligned}$$

in which, $\varvec{n}_e^{(i)}$ = $\varvec{F}_e\,\varvec{N}^{(i)}$ is the orientation of the fibres in the elastically deformed state and $\varvec{C}_e= \varvec{F}^T_e\,\varvec{F}_e$ is the right Cauchy-Green tensor associated with the elastic deformation. The dashpot of Fig. 11a is expressed linearly with the viscosity parameter $\eta _0$. A thermodynamically-consistent evolution equation detailed by Petiteau et al. (2013) is employed to describe the elastic deformation of the fibres and is defined in this case as follows:

11 $$\begin{aligned} \dot{\varvec{b}}_e=\varvec{L}\varvec{b}_e + \varvec{b}_e\varvec{L}^T-\frac{4}{\eta _0}\frac{\partial W_2}{\partial I_{4,e}}I_{4,e}\varvec{b}_e\left[ \left[ \varvec{n}_e\otimes \varvec{n}_e\right] -\frac{1}{3}I_{4,e}\varvec{I}\right] \end{aligned}$$

where $\varvec{b}_e$ = $\varvec{F}_e\,\varvec{F}^T_e$ is the elastic deformation left Cauchy-Green tensor and $\varvec{L} = \dot{\varvec{F}}\varvec{F}^{-1}$ is the velocity gradient tensor.

### One-dimensional formulation of the model

Now, the one-dimensional constitutive law will be formulated following the uniaxial tensile test condition of this study. For the muscularis propria, it is assumed that the collagen fibres, residing mainly in the longitudinal and circumferential directions, can be captured by two families of fibres whose mean orientations in the undeformed configuration are denoted by $\varvec{N}^{\left( 1\right) }$ and $\varvec{N}^{\left( 2\right) }$, as seen in Fig. 10. For the longitudinal samples, the direction vectors are simply:

12 $$\begin{aligned} \varvec{N}^{\left( 1\right) } = \begin{bmatrix} 1 &{} 0 &{} 0\\ \end{bmatrix}; \quad \varvec{N}^{\left( 2\right) } = \begin{bmatrix} 0 &{} 1 &{} 0\\ \end{bmatrix}. \end{aligned}$$

For uniaxial tension, the samples are loaded in only one direction, while the other two directions are unhindered, hence $\lambda _x$ = $\lambda$ for the longitudinal samples. In this model, as is with Holzapfel et al. (2000), it is assumed that the fibres are only active in tension, i.e., $I^{(i)}_{4}\ge 1$ and $I^{(i)}_{4,e}\ge 1$. It is also considered that the material is incompressible. Therefore, with the assumption of symmetry, the uniaxial tension deformation gradient tensor can be written as follows:

13 $$\begin{aligned} \begin{bmatrix}\varvec{F}\end{bmatrix} = \begin{bmatrix} \lambda &{} 0 &{} 0 \\ 0 &{} \lambda ^{-\frac{1}{2}} &{} 0 \\ 0 &{} 0 &{} \lambda ^{-\frac{1}{2}} \end{bmatrix} \end{aligned}$$

where $\lambda$ is the stretch as defined in Sect. 2.6. A Neo-Hookean SEF is used to model the matrix of the material and is described as:

14 $$\begin{aligned} W_0 = c_1[I_1 - 3] \end{aligned}$$

where $c_1$ is a stress-like material parameter. A classic Holzapfel model (Holzapfel et al. 2000) is used for the SEF of the fibres and is defined as follows:

15 $$\begin{aligned} W_1^{\left( i\right) }=\frac{k_1^{\left( i\right) }}{2k_2^{\left( i\right) }}[e^{ k_2^{\left( i\right) } [I_4^{\left( i\right) }-1]^2}-1] \end{aligned}$$

where $k_1 > 0$ is a stress-like material parameter and $k_2 > 0$ is a dimensionless parameter. For a review on a range of anisotropic, hyperelastic SEFs applicable for modelling soft tissues, readers are referred to Chagnon et al. (2015). For the elastic deformation of the fibres, a Kaliske (2000) SEF is used (with $n=3$) and is described as:

16 $$\begin{aligned} W_2^{\left( i\right) }=C_{2}^{\left( i\right) }[I_{4,e}^{\left( i\right) } - 1]^2 + C_{3}^{\left( i\right) }[I_{4,e}^{\left( i\right) } - 1] ^3 + C_{4}^{\left( i\right) }[I_{4,e}^{\left( i\right) } - 1] ^4 \end{aligned}$$

where $C_{2}$, $C_{3}$ and $C_{4}$ are stress-like material parameters. Using the definitions of Eqs. (4) and (10) with (13), the strain invariants can be written in terms of global stretch, $\lambda$, and the elastic stretch component, $\lambda _e$, for uniaxial tension as:

17 $$\begin{aligned} I_1 = \lambda ^2 + 2\lambda ^{-1}; \quad I^{(1)}_{4}= \lambda ^2; \quad I^{(2)}_{4}= \lambda ^{-1}; \quad I^{(1)}_{4,e} = \lambda _e^2; \quad I^{(2)}_{4,e} = \lambda _e^{-1}. \end{aligned}$$

The first PK stress tensor is then calculated from the Cauchy stress using $\varvec{P} = J \varvec{\sigma }\,\varvec{F}^{-T}$. The unknown hydrostatic pressure is solved using the knowledge that $P_z = 0$ for this test condition. Therefore, by inserting this and the definitions from (17), the one-dimensional first PK stress for the longitudinal samples becomes:

18 $$\begin{aligned} P_{x} = 2 \frac{\partial W_0}{\partial {I_1}}[\lambda - \lambda ^{-2}] + 2\chi ^{\left( 1\right) }\lambda ^{-1}\left[ \frac{\partial W_1^{\left( 1\right) }}{\partial {I_4^{\left( 1\right) }}}\lambda ^4 + \frac{\partial W_2^{\left( 1\right) }}{\partial {I_{4,e}^{\left( 1\right) }}}\lambda _e^4\right] , \end{aligned}$$

where the partial derivatives of the SEFs for the fibres and the matrix with respect to their invariants are as follows:

19 $$\begin{aligned} \frac{\partial W_0}{\partial {I_1}} = c_1; \quad \frac{\partial W_1^{\left( 1\right) }}{\partial {I_4^{\left( 1\right) }}} = \left[ \lambda ^2-1\right] k_1^{\left( 1\right) } e^{ k_2 [\lambda ^2-1]^2}; \end{aligned}$$

20 $$\begin{aligned} \frac{\partial W_2^{\left( 1\right) }}{\partial {I_{4,e}^{\left( 1\right) }}} = 2C_{2}^{\left( 1\right) }[\lambda _e^2 - 1] + 3C_{3}^{\left( 1\right) }[\lambda _e^2 - 1]^2 + 4C_{4}^{\left( 1\right) }[\lambda _e^2 - 1]^3. \end{aligned}$$

The first PK stress is now expressed in terms of $\lambda$ and can be compared to the experimentally obtained data. The same procedure is used to derive the one-dimensional equation for the circumferential direction, i.e. when $\varvec{N}^{(2)}$ is parallel to the axis of loading. The evolution equation of Eqn. (11) is used to attain the elastic deformation during loading and is written in its one-dimensional form in terms of $\lambda _e$ for uniaxial tension as:

21 $$\begin{aligned} \dot{\lambda }_e=\lambda _e\frac{\dot{\lambda }}{\lambda }-\frac{4}{3\eta _0}\frac{\partial W_2}{\partial I_{4,e}}\lambda _e^5 \end{aligned}$$

### Parameter identification and model validation

**Fig. 12:**
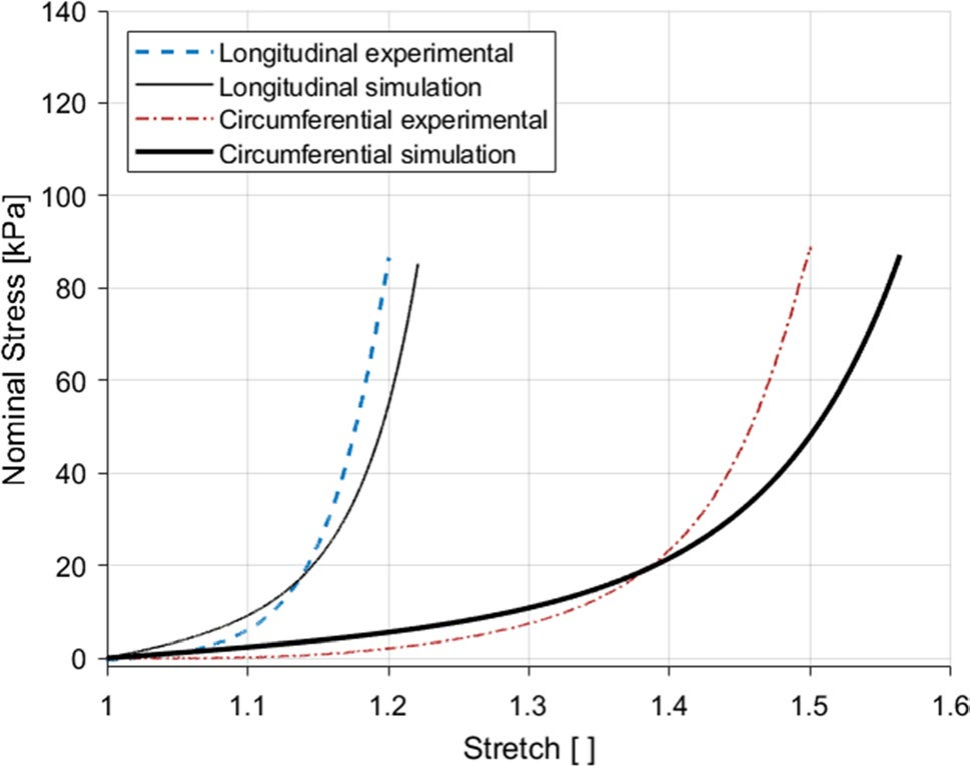
Identification of the hyperelastic parameters from the 1% s$^{-1}$ experimental results using the loading path of the second cycle of the final full stretch level for the longitudinal and circumferential directions

The first step in parameter identification was to isolate the hyperelastic portion of the experimental results. For this, the loading path closest to that of a preconditioned sample from the 1% ${\text{s}}^{-1}$ tests was used. Therefore, the loading path of the second cycle of the highest stretch level reached by each sample was extracted and used to identify only the hyperelastic parameters. The hyperelastic portion of the first PK stress equations for the longitudinal and circumferential directions were compared to the linear region of the experimental data to simultaneously fit the $c_1$ parameter using a manual slider. The $k_1^{(i)}$ and $k_2^{(i)}$ parameters were then obtained independently for each direction also by using a manual slider. The results of these fittings can be seen in Fig. 12, revealing a good simulation of the experimental data for both directions. The hyperelastic parameters, $c_1=1.32$  kPa, $k_1^{(1)}=13.4$  kPa, $k_1^{(2)}=3.36$  kPa, $k_2^{(1)}=6.85$ and $k_2^{(2)}=0.82$, are then kept frozen during the identification of the damage and viscous parameters.

**Fig. 13:**
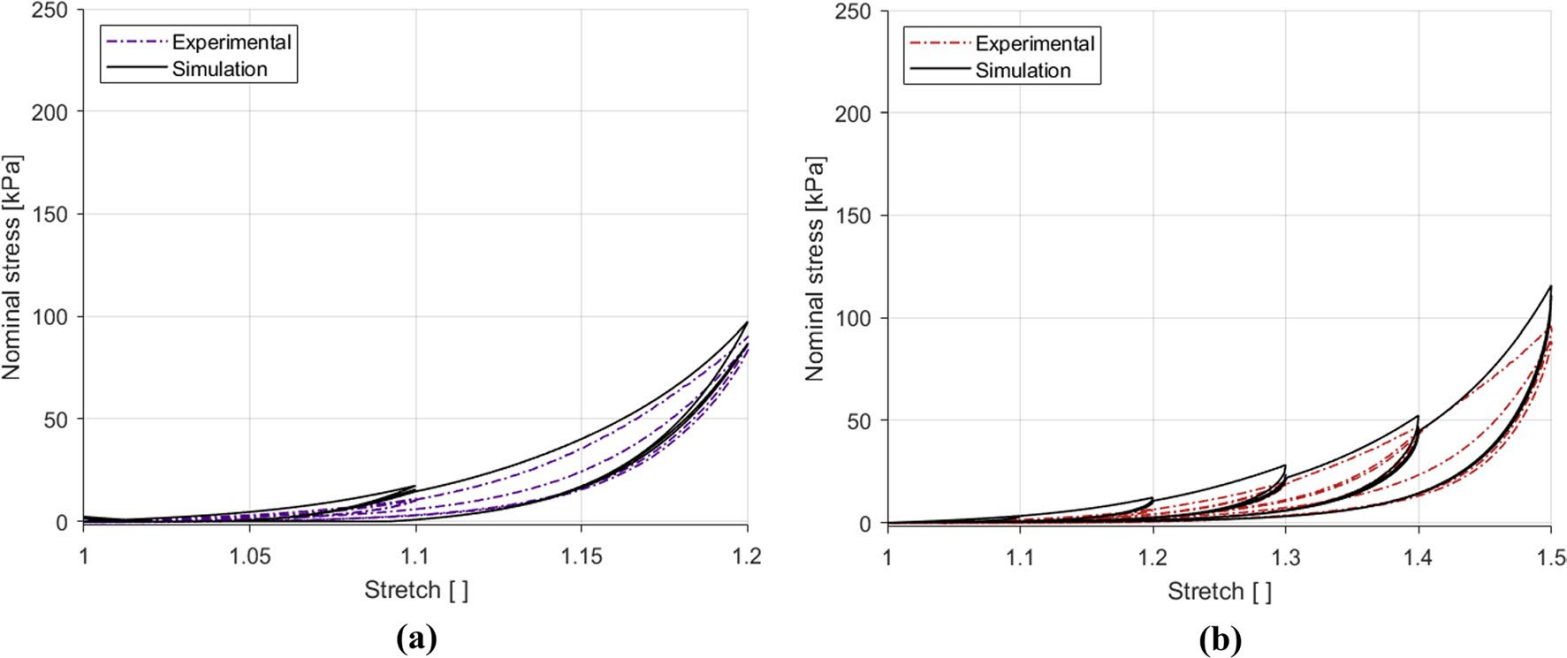
Parameter identification and modelling of the cyclic behaviour of the embalmed muscularis propria layer for the 1% ${\text{s}}^{-1}$ experimental results in the longitudinal direction (**a**) and the circumferential direction (**b**)

**Table 5:** A complete set of material parameter values of the visco-anisotropic damage model identified in a modularised way

	$c_1 [\text{kPa} ]$	$k_1 [\text{kPa} ]$	$k_2 [\text{kPa} ]$	$\eta _{\text{m}} [-]$	$\beta [-]$	$\eta _{0}$	$C_{2}[\text{kPa} ]$	$C_{3}[\text{kPa} ]$	$C_{4}[\text{kPa} ]$
$N^{(1)}$	1.32	13.4	6.85	2.16	1.20	2280	0.02	0.01	82.3
$N^{(2)}$		3.36	0.82	1.29	0.42	3309	0.02	1.14	3.00

**Fig. 14:**
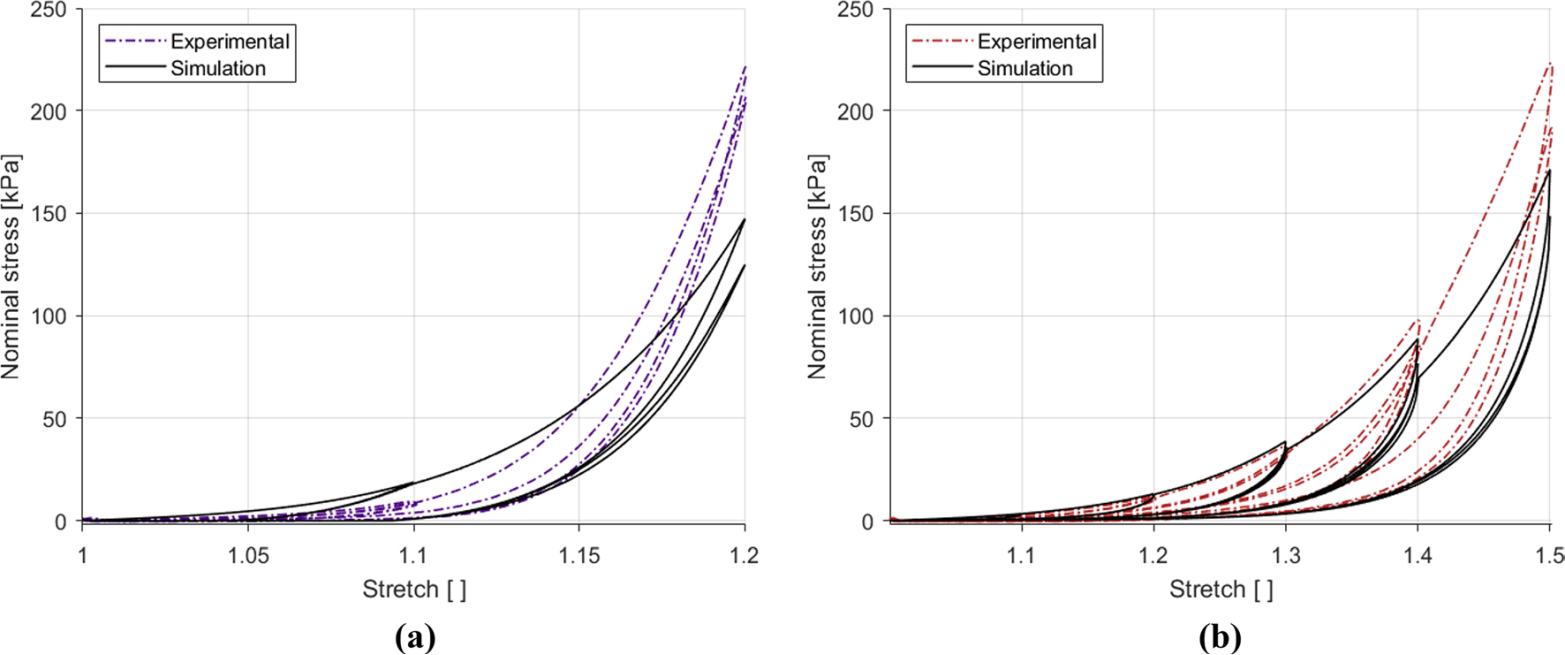
Parameter validation and modelling of the cyclic behaviour of the embalmed muscularis propria layer for the 10% ${\text{s}}^{-1}$ experimental results in the longitudinal direction (**a**) and the circumferential direction (**b**)

The cyclic data for the 1% s$^{-1}$ results in each direction were used to identify the viscous and damage parameters. For this, the lsqcurvefit function in MATLAB was utilised to obtain the parameters separately for the longitudinal and circumferential directions. The results of these fittings can be found in Fig. 13. The model provides a good fit for both directions, capturing well the nonlinearity and hysteresis of the experimental results. All the parameter values can be found in Table 5. The next step is to validate the model using experimental data that has not been used in the identification process. For this, the 10% s$^{-1}$ results for each direction were predicted. The results of this validation can be seen in Fig. 14. The model in the longitudinal direction, the results of which are depicted in Fig. 14a, simulates the general increase in the stiffness seen, but does not ideally predict the nonlinearity, steep gradient and reduced hysteresis seen at the higher strain rate. The prediction of the 10% s$^{-1}$ circumferential results simulates very well the behaviour up until the 1.4 stretch level, however past this point underestimates the maximum stress for this direction. All the model results do not adequately simulate the second cycle of each stretch level. The results overall, however, do well in modelling a good number of features of the stress-stretch response of the embalmed muscularis propria, including permanent deformations, hysteresis, nonlinearity, and predict well the effect of the increase in strain rate on the tissue’s behaviour, particularly in the circumferential direction.

## Discussion

The results of this study provide valuable insight into the material behaviour of the muscularis propria of the human oesophagus. The embalmed muscular layer displayed hyperelastic properties, with a later onset of strain hardening in the circumferential direction compared to the longitudinal direction. The layer also displayed anisotropy in terms of stiffness, where the longitudinal direction was stiffer than the circumferential for both strain rates. These findings are in line with those of comparable animal studies by Sommer et al. (2013), Stavropoulou et al. (2012), and Yang et al. (2006b). The histological analysis revealed the collagen fibres of the circular and longitudinal muscular layers to align mainly with their respective muscle fibres, but found that the majority of collagen of the muscularis propria was situated at the junction of the two muscular layers and was orientated longitudinally. These histological findings are in line with similar analysis by Stavropoulou et al. (2009) who studied the rabbit oesophagus. As collagen is associated with the tensile strength of soft tissues (Aziz et al. 2016), the anisotropy of the layer is thought mainly to be due to the preferential alignment of collagen within the layer.

The nonlinearity of the muscular layer was captured well by the hyperelastic model, for which a physically-driven micromechanical model was used. This type of model is preferential to a purely phenomenological hyperelastic model as it aids in establishing how the microstructure of the oesophagus relates to its stress-strain response and physiological function. An extension to the classic Holzapfel model developed by Gasser et al. (2006) takes into account the dispersion of the fibres within a material. This method, however, was not adopted here due to (1) the histological findings showing that the collagen fibres predominantly reside in the longitudinal and circumferential directions and (2) the lack of means to establish the fibre dispersion experimentally. Although some degree of fibre dispersion is inherent to biological tissues, the classic Holzapfel model is more suited to the type of histological data collected in this study. In addition, if a dispersion model were to be implemented without any experimentally determined fibre dispersion data, a phenomenological fitting of the dispersion parameter would be required, which would result only in increasing the number of parameters of the model without providing any insight into the physical composition of the layer (Chagnon et al. 2015). Overall, the parameters of the classic Holzapfel model correlated well with the histological findings of the study in that the fibre stiffness parameter was approximately four times higher for the longitudinal direction than the circumferential direction.

Nonlinearity of oesophageal tissue has been related to its physiological function, wherein the wall displays compliance at low strains to accommodate for the swallowing process, but stiffens at high strains in order to prevent over-dilatation (Mir et al. 2016). This provides explanation of the nonlinearity and lower initial stiffness seen in the circumferential direction, but fails to consider the material behaviour observed in the longitudinal direction. Physiologically, contraction of the longitudinal muscle fibres causes the oesophagus to shorten. Local shortening increases the thickness of the oesophageal wall and reduces the work of the circular muscle fibres when contracting to transport the fluid bolus (Brasseur et al. 2007). The stiffness of the longitudinal direction under tension, therefore, is thought to resist over-extension in the longitudinal direction during passage of the bolus, so as to allow the longitudinal muscle fibres to effectively carry out their primary role of local shortening during peristalsis.

Viscoelasticity of the muscular layer was observed in both directions, with larger hysteresis in the longitudinal direction than the circumferential direction up until 1.2–1.3 stretch. After this point, across both strain rates, the longitudinal samples rupture, undergoing irreversible structural damage, while the hysteresis of the circumferential samples increase as the direction continues to be compliant at higher deformations. As well as tensile strength, collagen fibres are attributed to a notable proportion of the viscoelastic behaviour of soft tissues (Li et al. 2005; Yang et al. 2006a). The greater hysteresis and strain rate-dependency seen at the lower stretches in the longitudinal direction are hypothesised to be due to the greater collagen content in this direction, while the higher stressibility and increase in strain rate effects and hysteresis as the stretch increases in the circumferential direction could be due a later onset of fibre activation. Yet, it can be seen that the viscosity parameter of the model was greater for the circumferential direction than the longitudinal direction. The viscoelastic model of the study underestimated the change in stiffness of the longitudinal direction when predicting its response at the higher strain rate. This suggests that the viscosity of the longitudinal direction may be more nonlinear than the circumferential direction, who’s increase in stiffness was sufficiently captured by the linear viscoelastic model used here. Therefore, to more accurately model the time-dependent stress–strain response of the oesophageal muscular layer, a nonlinear viscoelastic model should be considered in the future.

The maximal stress observed in the experimental data for the 10% ${\text{s}}^{-1}$ tests in the longitudinal direction was similar to that established by Egorov et al. (2002), as stated in Sect. 1, who studied only the longitudinal direction of the human oesophagus. Egorov et al. (2002) studied the organ with its layers intact and used tissue from fresh cadavers. As the maximal stress value is very similar for the longitudinal direction when comparing an individual layer to an intact wall, the higher stiffness seen in this study than expected could be due to the embalming process. Embalming is considered to predominantly affect the collagen of soft tissues, wherein formalin solution causes collagen cross-links to form (Fessel et al. 2011). This, logically, would imply an embalming-induced increase in stiffness. Within literature, however, the effect of formalin on the mechanical properties of soft tissues is inconclusive; some studies have found the stiffness of tissues to increase (Hohmann et al. 2019), while others found the stiffness to decrease (Girard et al. 2019). Girard et al. (2019) found that embalming decreased the mechanical properties of the human bile duct by 80% in the longitudinal direction (the more collagen-dense direction) and 40% in the circumferential direction compared to the fresh equivalent, while Hohmann et al. (2019) found the Young’s modulus of the human upper biceps tendon to be approximately 20 times higher for the embalmed tendon compared to the fresh. Despite conflicting findings in literature, it is hypothesised that in the current study embalming caused the stiffness of the tissue to increase due to the relation of the results to the findings of Egorov et al. (2002). That being said, the relationship between the longitudinal and circumferential directions remains consistent with the other anisotropic, ex vivo study looking into the human oesophagus by Vanags et al. (2003), and also with similar layer-dependent, anisotropic studies conducted on animal oesophagi (Sommer et al. 2013; Yang et al. 2006b; Stavropoulou et al. 2012). It is worthy to note that the Young’s moduli of this study, according to the findings of Vanags et al. (2003), might be greater here than for younger oesophageal tissue due to the high ages of the patients tested, as stated in Sect. 3.2 .

Variation when testing human soft tissues is prevalent, with many variables potentially affecting their mechanical properties and therefore the mechanical data collected. These include, but are not exclusive to, age, sex, and health of the patient, as well as experimental variables such as time since explantation, sample thickness due to variation in biological composition throughout the tissue, temperature and moisture of the samples, and storage technique. In the current study, variability in terms of rupture stretch, rupture stress and stiffness were observed between different tests of the same direction and strain rate. Some of these differences may be attributed to the variations seen in sample dimensions. While measures were taken to reduce discrepancies, for instance by obtaining samples primarily from the inferior section of the thoracic region wherein the longitudinal muscle fibres are more evenly distributed (as outlined in Sect. 2.1), natural variation was still present, leading to the range of sample thicknesses seen in Table 3. The differences amongst the width of the samples are attributed to the human error associated with cutting samples by hand. Human error was also potentially present in the measuring of the width and thickness of the samples. To reduce the risk of this, measurements were taken by a single person, and to reduce any effect, three separate measurements were taken per dimension along the sample and an average was used.

## Conclusion

This study has presented a unique insight into the layer-specific properties of the human oesophagus through the investigation of its embalmed muscular layer. Overall, the muscularis propria demonstrated anisotropy with distinct behaviour in each direction, hyperelasticity through its nonlinear response, viscoelasticity in the form of hysteresis and a strain rate-dependency, and damage through the occurrence of stress-softening and permanent set. This behaviour was captured fairly well by the matrix-fibre model of the study, which simulates particularly well the response of the layer in the circumferential direction. Although ideally fresh tissue would be used to characterise soft tissues, the results presented here give an idea of what is to be expected from the behaviour of the fresh human muscularis propria, including its approximate dissipation, nonlinearity, strain rate-dependency, and the anisotropy of these. In addition, once the COVID-19 restrictions have been lifted, the layer-specific fresh tissue properties will be established and, in a forthcoming contribution, compared with the embalmed results presented here. These results will establish the effects of embalming as a preservation technique on the human oesophagus; the knowledge of which can be used in applications such as surgical training for medical students, for which embalmed cadavers are often used instead of fresh (Hayashi et al. 2016; Kennel et al. 2018). Further to this, through understanding how embalming affects the tissue’s microstructure and mechanical behaviour, and through comparing these with fresh tissue, one may establish more confidently how each constituent of the soft tissue contributes to its various material phenomena. Moreover, numerical modelling will be carried out on the fresh tissue results to provide more physiologically-relevant parameters, with a Finite Element implementation of this in the plans for future work.
